# Cetaceans Have Complex Brains for Complex Cognition

**DOI:** 10.1371/journal.pbio.0050139

**Published:** 2007-05-15

**Authors:** Lori Marino, Richard C Connor, R. Ewan Fordyce, Louis M Herman, Patrick R Hof, Louis Lefebvre, David Lusseau, Brenda McCowan, Esther A Nimchinsky, Adam A Pack, Luke Rendell, Joy S Reidenberg, Diana Reiss, Mark D Uhen, Estel Van der Gucht, Hal Whitehead

## Abstract

A group of eminent cetacean researchers respond to headlines charging that dolphins might be "flippin' idiots". They examine behavioural, anatomical and evolutionary data to conclude that the large brain of cetaceans evolved to support complex cognitive abilities.

The brain of a sperm whale is about 60% larger in absolute mass than that of an elephant. Furthermore, the brains of toothed whales and dolphins are significantly larger than those of any nonhuman primates and are second only to human brains when measured with respect to body size [1]. How and why did such large brains evolve in these modern cetaceans? One current view of the evolution of dolphin brains is that their large size was primarily a response to social forces—the requirements for effective functioning within a complex society characterized by communication and collaboration as well as competition among group members [2–4]. In such a society, individuals can benefit from the recognition of others and knowledge of their relationships and from flexibility in adapting to or implementing new behaviors as social or ecological context shifts. Other views focus on the cognitive demands associated with the use of echolocation [5–7].

Recently, Manger [8] made the controversial claim that cetacean brains are large because they contain an unusually large number of thermogenic glial cells whose numbers increased greatly to counteract heat loss during a decrease in ocean temperatures in the Eocene-Oligocene transition. Therefore, he argues, cetacean brain size could have evolved independently of any cognitive demands and, further, that there is neither neuronal evidence nor behavioral evidence of complex cognition in cetaceans. These claims have garnered considerable attention in the popular press, because they challenge prevailing knowledge and understanding of cetacean brain evolution, cognition, and behavior.

We believe that the time is ripe to present an integrated view of cetacean brains, behavior, and evolution based on the wealth of accumulated and recent data on these topics. Our conclusions support the more generally accepted view that the large brain of cetaceans evolved to support complex cognitive abilities.

## The Origins and Evolution of Large Brains in Odontocetes

**Figure 1:**
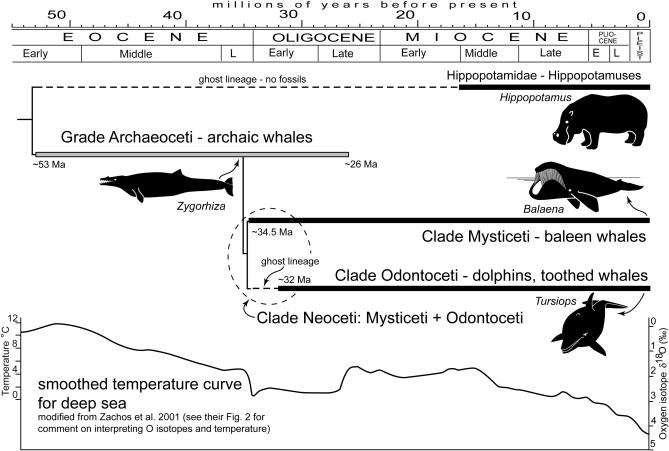
Relationships among Odontoceti and Mysticeti, between Neoceti and Archaeoceti, and higher level taxa of Whippomorpha (Cetacea + Hippopotamidae) Note that within Cetacea, the only ghost lineage (any length of time missing fossils as inferred from the phylogeny) is a short gap at the origin of Odontoceti. There is a large ghost lineage between Hippopotamidae and the base of Cetacea. The temperature curve shows a smoothed record for the deep sea, in turn a proxy for global climate.

The cetaceans arose from artiodactyls (even-toed ungulates) early in the Eocene approximately 55 million years ago (Figure 1) [9,10]. The earliest cetaceans, archaeocetes, were not highly encephalized; rather there was a significant increase in relative brain size in odontocetes (toothed whales, including dolphins) during their initial radiation in the late Eocene–early Oligocene transition [11]. This dramatic increase in relative brain size involved a substantial decrease in body size with a concurrent, more moderate, increase in brain size.

As Manger correctly points out, there is evidence for oceanic cooling during late Eocene-Oligocene times (Figure 1) [12]. Odontocete bodies actually got smaller during that time, whereas, generally, cooler climates induce increases in body size [e.g., 13], because larger animals lose relatively less heat to the environment. Moreover, cetaceans were already well above the threshold for body size to deal with oceanic cooling [14]. Therefore, there was no need for odontocetes to respond to these temperature decreases with either change in body size or brain size. Thus, such changes in brain size (and body size) in odontocetes were likely due to factors other than oceanic temperature change.

Concurrent with changes in relative size, the brain reorganized into a form with relatively larger cerebral hemispheres and overall greater similarity to that of modern cetaceans [11]. Tentative evidence also suggests concomitant changes in cranial architecture and ear structure to support echolocation [15]. Although the selection pressure that drove the decrease in body size is unknown, smaller animals would have experienced changes in their ecology (e.g., predation risk) that may have driven further behavioral changes. This may indicate that the large brains of early odontocetes were used, at least partly, for processing this entirely new sensory mode that evolved at the same time as these anatomical changes and perhaps for integrating this new mode into an increasingly complex behavioral ecological system.

## Contemporary Cetacean Neuroanatomy

The common ancestor of cetaceans and primates lived over 95 million years ago [16], and cetacean brains have been on an independent evolutionary trajectory from other mammals for close to 55 million years [17]. During that time, cetacean brains evolved a unique combination of features that are different in many respects from primate brains.

The cetacean neocortex was once viewed as relatively homogeneous in cellular architecture, regionally unspecialized, and lacking organizational complexity. It was thought to have poorly differentiated neuronal morphology, low numbers of neurons and cortical areas, and an indistinct prefrontal cortex. This view of cetacean neocortex harks back to an earlier era when a few authors who considered dolphins rather unintelligent saw little in the neuroanatomy, not surprisingly, to refute that view [18,19]. This perspective influenced later thinking about cetacean brains and led to the “initial brain” hypothesis of cetacean neocortical evolution [20] that asserted cetacean neocortex was primitive. However, modern neuroanatomical techniques convincingly demonstrate that the cetacean neocortex has a degree of regional parcellation comparable to that of many terrestrial mammals (see Box 1) [21,22]. There is certainly no evidence that the “cetacean scheme” is incapable of supporting complex processing similar to that in primates and other mammals.

**Box 1. Complexity in the Cetacean Neocortex**

The cetacean neocortex surpasses in gyrification all other mammals, including humans [61,62], as seen on panels A and B showing parasagittal sections through the brains of a bottlenose dolphin (A) and a humpback whale (B, anterior is to the left). The cetacean neocortex comprises limbic, paralimbic, and supralimbic regions [63]. The cetacean neocortex is thin, and it has a prominent thick layer I, which is far more cellular than in terrestrial species. It also displays large inverted neurons in the cell-dense layer II, and very large pyramidal neurons arranged in clusters of variable size at the border between layers III and V. Layers III and VI vary considerably in thickness and cellular density across regions [21,22]. The cetacean neocortex appears agranular due to a lack of layer IV. Studies of neocortical cytoarchitecture in several cetacean species reveal clearly identifiable cortical domains and regional complexity as seen in primates and carnivores [21,22,64–66]. The photomontages in (C) show examples of the region likely to correspond to the primary visual cortex in the humpback whale, the Cuvier's beaked whale, the beluga whale, the dwarf sperm whale, and the striped dolphin. Note the alternating neuronal modules, characteristic of this region, forming columns and patches of neuropil in layers V and VI. The absence of layer IV, the thickness of layers I and layer VI patterns may mean that thalamocortical projections of cetaceans rely on a very different wiring scheme than in terrestrial species. In fact, mysticetes exhibit striking cortical modules in layer II of vast expanses of the occipital cortex ([D], arrowheads), that are not observed in odontocetes (or other mammals) in this location, but are reminiscent of those seen in the entorhinal cortex of mammals and in the insula of toothed whales. These neuron clusters may represent a strategy to optimize intrahemispheric connectivity in the very large brains of mysticetes [22]. In the box figure, cortical layers are indicated by Roman numerals; wm, white matter (C, D). Scale bars: (D), 400 μm; (C), except for S. coeruleoalba, 250 μm; (A), 1.2 cm; (B), 3.5 cm.

**Figure:**
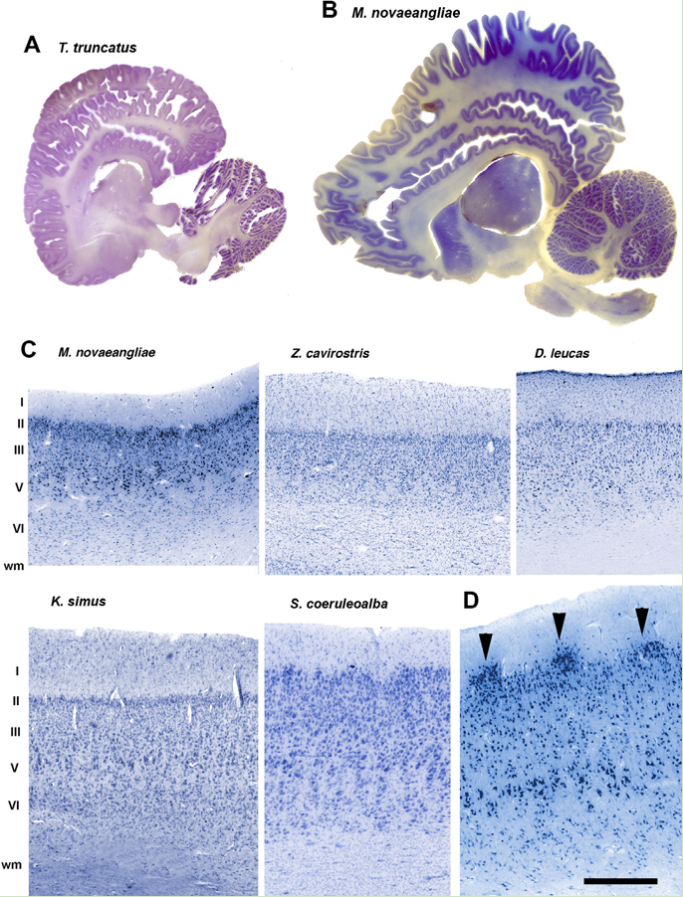

**Figure 2:**
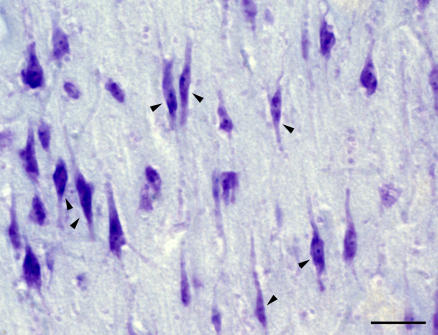
Spindle Cells in the Humpback Whale Anterior Cingulate Cortex A large number of spindle cells (arrowheads) are found in the anterior cingulate and insular and frontopolar cortices. They exhibit an elongate morphology with clearly visible apical and basal dendrites, and frequent grouping in clusters. Scale bar = 100 μm.

Likewise, there is no reason to expect that cetacean and primate prefrontal cortical analogs would be, in fact, located in the same region of the brain. However, the expansion of the insular and cingulate cortices in cetaceans is consistent with high-level cognitive functions—such as attention, judgment, intuition, and social awareness—known to be associated with these regions in primates [23]. This view is further supported by the observation that the anterior insular and anterior cingulate cortex in cetacean species having the largest brains exhibit a large number of large layer V spindle neurons [22] (Figure 2), similar to those originally reported to be unique to humans and great apes [24,25]. These particular neurons are considered to be responsible for neural networks subserving aspects of social cognition [23].

The cetacean neocortex is also characterized by a high ratio of glial cells to neurons, consistent with the general pattern found in other mammals, where neuron density decreases with absolute brain size, probably to maintain certain properties of neural transmission. “Glia” include several distinct cell populations, including: (1) oligodendrocytes, which provide myelin for axons or “white matter;” (2) astrocytes, which have several roles and predominate in the gray matter; and (3) microglia, immune cells which are not embryologically related to other glia or neurons. Given their vastly different roles, it is important to know which is being counted to interpret the functional significance of a high glial cell/neuron ratio in cetaceans. If, for instance, a high glial cell/neuron ratio is due to an increase in oligodendrocytes, this would be consistent with previous observations that as brains get larger, the white matter increases proportionally more than the gray matter [26]. In fact, recent imaging studies show that it is precisely by a greater proportion of white matter that humans can be distinguished from apes and monkeys [27,28]. Moreover, growing evidence demonstrates that astrocytes contribute to the modulation and coordination of neural activity in the brain [29–31]. Therefore, despite Manger's argument, a high glia cell/neuron ratio is consistent with the increased needs of complex brains for rapid communication and synaptic efficiency.

## Cetacean Cognition and Behavior in the Laboratory

The preceding description of cetacean brains reveals not only their large absolute and relative size but also underscores a structural complexity that could support complex information processing, allowing for intelligent, rational behavior. There is considerable behavioral data to support that assumption.

Laboratory studies of bottlenose dolphins have documented various dimensions of their intellectual abilities. These include an understanding of symbolic representations of things and events (declarative knowledge); an understanding of how things work or how to manipulate them (procedural knowledge); an understanding of the activities, identities, and behaviors of others, (social knowledge); and an understanding of one's own image, behavior, and body parts (self knowledge) [reviewed in 32]. All these capabilities rest on a strong foundation of memory; investigations have demonstrated that bottlenose dolphin auditory, visual, and spatial memory are accurate and robust [33–36].

Learning, remembering, and innovation can be life-saving cognitive tools in a challenging environment. The flexible and diverse learning capabilities of dolphins are well documented, including, for example, the learning of a variety of types of abstract rules [37,38] and the spontaneous understanding and execution of instructions from televised trainers [39]. Learning of an imposed language is perhaps the most challenging cognitive task that dolphins have faced in the laboratory. Dolphins learned to understand not only the semantic features of artificial gestural and acoustic languages, but also the syntactic features [40]. Learning of complex syntactic structures or decoding of anomalous structures was often achieved through inference, rather than through explicit instruction [41].

Dolphins spontaneously learn associations between sounds and temporally paired events [42] and demonstrate extensive imitative abilities for sounds and for behaviors (see Box 2) [42, 43–45]. Dolphins can develop a concept of mimicry—copying an observed behavior or sound if given a symbolic instruction to do so. Dolphins are the only mammal, other than humans, shown capable of extensive and rich vocal and behavioral mimicry. Indeed the evidence that bottlenose dolphins are capable of imitation, one of the highest forms of social learning, is so strong that a leading primatologist has concluded that they “ape better than apes” [46].

**Box 2. Imitation in Dolphins**

Imitation is an important type of social learning that can readily lead to stable cultures. While it is clear that many cetaceans are natural mimics, executing synchronous motor behaviors, such as “porpoising” in unison, and spontaneously imitating sounds, including the whistles of others, imitation is a complex multidimensional ability that is most intimately studied in the laboratory. Bottlenose dolphin abilities for both arbitrary vocal and motor imitation were demonstrated at the Kewalo Basin Marine Mammal Laboratory in Honolulu. Vocal imitation was investigated by broadcasting electronically generated “model” sounds underwater into a dolphin's habitat [43]. In response, the dolphin vocalized into a hydrophone. Figure A in this box shows spectrograms of each of nine model sounds and the resulting imitation. The arrow points to the beginning of the dolphin's imitation. A variety of different waveforms were imitated accurately; the imitations of sounds G and H show spontaneous octave generalization, the imitation occurring precisely an octave above (G) or an octave below (H) the model sound. Octave generalization is a rare ability that, for example, has not been elicited from songbirds.

**Figure A:**
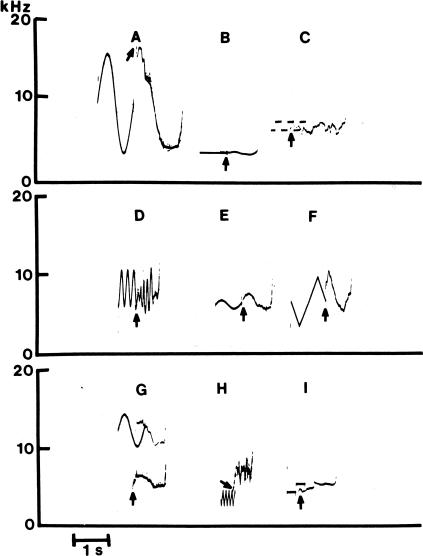
Spectrograms of each of nine model sounds and the resulting imitation. The arrow points to the beginning of the dolphin's imitation.

Social motor imitation was demonstrated first by having two dolphins side by side with a partition between them that allowed the dolphins to see each other but not their respective trainers. The “demonstrator” dolphin was instructed gesturally by its trainer to perform one of many possible behaviors, including its own self-chosen behavior. Then, the “imitator” dolphin was instructed by its trainer to either “mimic” the demonstrated behavior or to perform another behavior. Both dolphins successfully imitated familiar and novel modeled behaviors. This ability generalized easily to imitating human behaviors demonstrated either at poolside (Figure B) or on a television monitor placed behind an underwater window. Motor mimicry also extended to self-imitation, the imitation of one's own previous behavior. No nonhuman animal has shown the levels of diversity, flexibility, and cognitive control of imitative skill demonstrated in bottlenose dolphins [44].

**Figure B:**
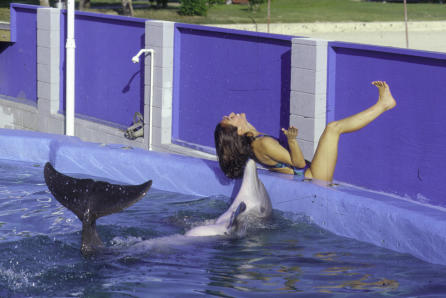
Dolphin imitates the behavior of a human by using its tail as an analogy for a leg.

Social knowledge includes awareness of the indications of another. Dolphins readily learn to understand the significance of human pointing gestures and head gaze [47–49]. They attend not only to the direction in which the human points or gazes, but also to the object of regard [50]. Dolphins can also attend to a target being echoically interrogated by another dolphin by “eavesdropping” on the returning echoes [51]. Dolphins echolocate by orienting both their body and their narrow-beam echolocation signal in a particular direction, which may be a rough analog to arm and hand directional pointing by humans [47]. Additionally, dolphins can use their rostrums and body alignment to point and direct a human swimmer to an object or place of interest [52] and monitor whether the human receiver is attending to them [52,53].

**Figure 3:**
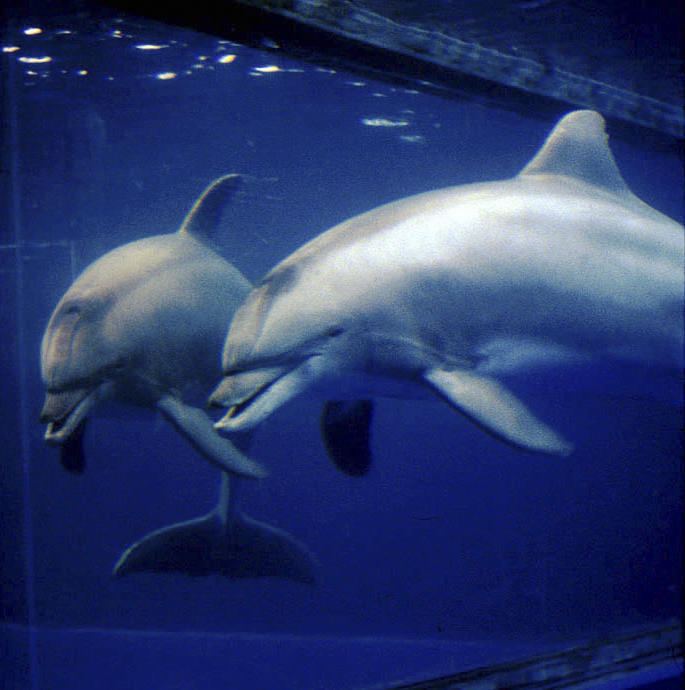
One of Two Bottlenose Dolphins That Passed the Mark Test, Thus Demonstrating Mirror Self-Recognition (Photo credit: Diana Reiss, Wildlife Conservation Society)

Self-knowledge, including self-awareness, enables one to develop a self-image and monitor and evaluate one's own behaviors. Dolphins recognize themselves in a mirror [54] (Figure 3), a rare ability previously demonstrated in the great apes and humans ([54] for a review) and, recently, in elephants [55]. Mirror self-recognition not only indicates an ability to correctly interpret information in a mirror as oneself but also demonstrates an individual's motivation to use the mirror as a tool to view one's own body. Dolphins are also aware of their own behaviors, able to understand and act on gestural instructions to repeat or not repeat a previously performed behavior, or to monitor self-produced bubble rings [56–58], Dolphins also reveal conscious awareness and conscious control of their own body parts, using them in specific and often novel ways as directed by gestural instructions [59]. Finally, dolphins demonstrate awareness of their own knowledge states, i.e., metacognition, by indicating their certainty or uncertainty about which of two sounds is of higher pitch [60].

## Cetacean Cognition and Behavior in the Wild

Beyond knowing what cetaceans can do with their large complex brains, it's equally important to ask what they do naturally. Long-term field research has shown that dolphins live in large complex groups with highly differentiated relationships that include long-term bonds, higher-order alliances, and cooperative networks [61–62] that rely on learning and memory. Some of the complexities typical of within-group primate alliances, such as individuals switching sides in different social contexts, are also seen among bottlenose dolphins. Moreover, “alliances of alliances,” observed in bottlenose dolphins, are rare outside of our own species, even among old world monkeys and apes [3]. There is also evidence that individual role taking has emerged in dolphin societies to facilitate cooperative relationships [63] and decision-making processes [64,65].

Field studies have documented impressive cultural learning of dialects, foraging sites, and foraging and feeding strategies in cetaceans. Culture, the transmission of learned behavior, is one of the attributes of cetaceans that most sets them apart from the majority of other nonhuman species [66] and is likely underpinned by advanced social learning abilities. Cultural attributes have been identified in many species of cetaceans but principally in those best studied: the bottlenose dolphin, the killer whale, the sperm whale, and the humpback whale [66]. One of the most distinctive elements of cetacean culture is multiculturalism—groups with different cultures using the same habitat—which is known in bottlenose dolphins, humpback whales, killer whales, and sperm whales. For example, killer whale populations of the eastern North Pacific are structured into several social tiers, which possess distinctive cultural attributes in vocal, social, feeding, and play behavior [67,68].

Social complexity and culture in cetaceans are arguably dependent on a complex and flexible communication system, encompassing vocal, visual, tactual, and possibly chemical signals [69]. There are differences across cetaceans in their sound production mechanisms. Odontocetes (primarily high-frequency producers, echolocating) and mysticetes (primarily low-frequency producers, non-echolocating) exhibit radically divergent nasal, laryngeal, and hyoid anatomy [70–74]. Cetaceans also supplement their repertoire of vocal signals with visual cues (e.g., changes in body posture), tactile behaviors (e.g., flipper touching, teeth raking), and nonvocal auditory behaviors (e.g., breaching, lob tailing). The temporal sequencing of these latter nonvocal communicative events can be highly structured, demonstrating a complex and diverse nonvocal communication system [64,75].

Dolphins produce several different whistle types and sounds. Evidence also shows that the sequential order of whistle production is an important feature of their communication system [76,77]. Extensive fieldwork has shown that cetacean call types exhibit enormous variation [78,79], evolve over time [80], and are used differently across social groups [81]. In some cases, the variation is so pronounced that other species have learned to use it in judging predation risk [82]. In bottlenose dolphins, there is evidence that this variation is the basis for a referential identity-labeling system [83].

Cultural learning of behaviors may proceed through motor imitation or perhaps even through direct teaching (pedagogy), as may be the case for killer whale calves “instructed” in beach capture of pinnipeds by their mothers [66,84]. Vocal imitation also occurs, such as the development of dialects among killer whale family groups [78–80, 85]. The close synchrony seen among wild dolphins is a form of imitative behavior that may serve in part to express their affiliation [86]. Tool use, which is a measure of intelligence that correlates with relative brain size in primates [87] and birds [88], is also documented in dolphins, who use sponges to probe into crevices for prey and appear to transmit the technique culturally [89].

## Conclusion

Evidence from various domains of research demonstrates that cetacean brains underwent elaboration and reorganization during their evolution with resulting expansion of the neocortex. Cortical evolution, however, proceeded along very different lines than in primates and other large mammals. Despite this divergence, many cetaceans evince some of the most sophisticated cognitive abilities among all mammals and exhibit striking cognitive convergences with primates, including humans. In many ways, it is because of the evolution of similar levels of cognitive complexity via an alternative neuroanatomical path that comparative studies of cetacean brains and primate brains are so interesting. They are examples of convergent evolution of function largely in response, it appears, to similar societal demands.

Returning to Manger, his controversial claim is reminiscent of the conclusion reached about bees by physicists and mathematicians in the 1930s—that the anatomical structure of bees and the known principles of flight indicate that bee flight is impossible [90]. Rightfully oblivious to Manger's contentions, cetaceans continue to provide an enormous body of empirical evidence for complex behavior, learning, sociality, and culture.

## References

[pbio-0050139-b001] Marino L (1998). A comparison of encephalization between odontocete cetaceans and anthropoid primates. Brain Behav Evol.

[pbio-0050139-b002] Herman LM, Herman LM (1980). Cognitive characteristics of dolphins. Cetacean behavior: Mechanisms and functions.

[pbio-0050139-b003] Connor RC, Smolker RA, Richards AF, Harcourt AH, de Waal FBM (1992). Dolphin alliances and coalitions. Coalitions and alliances in animals and humans.

[pbio-0050139-b004] Connor RC (2007). Complex alliance relationships in bottlenose dolphins and a consideration of selective environments for extreme brain size evolution in mammals. 10.1098/rstb.2006.

[pbio-0050139-b005] Jerison HJ, Schusterman RJ, Thomas JA, Wood FG (1986). The perceptual world of dolphins. Dolphin cognition and behavior: a comparative approach.

[pbio-0050139-b006] Ridgway SH, Schusterman RJ, Thomas JA, Wood FG (1986). Physiological observations on dolphin brains. Dolphin cognition and behavior: a comparative approach.

[pbio-0050139-b007] Wood FG, Evans WE, Busnel R, Fish J (1980). Adaptiveness and ecology of echolocation in toothed whales. Animal sonar systems.

[pbio-0050139-b008] Manger PR (2006). An examination of cetacean brain structure with a novel hypothesis correlating thermogenesis to the evolution of a big brain. Biol Rev.

[pbio-0050139-b009] Fordyce RE, Prothero DR, Ivany LC, Nesbitt E (2003). Cetacean evolution and Eocene-Oligocene oceans revisited. From greenhouse to icehouse. The marine Eocene-Oligocene transition.

[pbio-0050139-b010] Geisler JH, Uhen MD (2005). Phylogenetic relationships of extinct Cetartiodactyls: Results of simultaneous analyses of molecular, morphological, and stratigraphic data. J Mamm Evol.

[pbio-0050139-b011] Marino L, McShea D, Uhen MD (2004). The origin and evolution of large brains in toothed whales. Anat Rec.

[pbio-0050139-b012] Zachos J, Pagani M, Sloan L, Thomas E, Billups K (2001). Trends, rhythms, and abberations in global climate 65 Ma to present. Science.

[pbio-0050139-b013] Millien V, Lyons SK, Olson L, Smith FA, Wilson AB (2006). Ecophenotypic variation in the context of global climate change: Revisiting the rules. Ecol Lett.

[pbio-0050139-b014] Downhower JF, Blumer LS (1988). Calculating just how small a whale can be. Nature.

[pbio-0050139-b015] Fleischer G (1976). Hearing in extinct cetaceans as determined by cochlear structure. J Paleontol.

[pbio-0050139-b016] Kumar S, Blair Hedges S (1998). A molecular timescale for vertebrate evolution. Nature.

[pbio-0050139-b017] Gingerich PD, Uhen MD (1998). Likelihood estimation of the time of origin of cetaceans and the time of divergence of cetaceans and Artiodactyla. Paleo-electronica.

[pbio-0050139-b018] Bianchi V (1905). Il mantello cerebrale del delfino (Delphinus delphis).

[pbio-0050139-b019] Kesarev VS (1971). The inferior brain of the dolphin. Soviet Sci Rev.

[pbio-0050139-b020] Glezer II, Jacobs MS, Morgane PJ (1988). Implications of the ‘initial brain’ concept for brain evolution in Cetacea. Behav Brain Sci.

[pbio-0050139-b021] Hof PR, Chanis R, Marino L (2005). Cortical complexity in cetacean brains. Anat Rec.

[pbio-0050139-b022] Hof PR, Van der Gucht E (2007). The structure of the cerebral cortex of the humpback whale, Megaptera novaeangliae (Cetacea, Mysticeti, Balaenopteridae). Anat Rec.

[pbio-0050139-b023] Allman JM, Watson KK, Tetreault NA, Hakeem AY (2005). Intuition and autism: A possible role for Von Economo neurons. Trends Cogn Sci.

[pbio-0050139-b024] Nimchinsky EA, Vogt BA, Morrison JH, Hof PR (1995). Spindle neurons of the human anterior cingulate cortex. J Comp Neurol.

[pbio-0050139-b025] Nimchinsky EA, Gilissen E, Allman JM, Perl DP, Erwin JM (1999). A neuronal morphologic type unique to humans and great apes. Proc Natl Acad Sci U S A.

[pbio-0050139-b026] Zhang K, Sejnowski TJ (2000). A universal scaling law between gray matter and white matter of cerebral cortex. Proc Natl Acad Sci U S A.

[pbio-0050139-b027] Schenker NM, Desgouttes AM, Semendeferi K (2005). Neural connectivity and cortical substrates of cognition in hominoids. J Hum Evol.

[pbio-0050139-b028] Schoenemann PT, Sheehan MJ, Glotzer LD (2005). Prefrontal white matter volume is disproportionately larger in humans than in other primates. Nat Neurosci.

[pbio-0050139-b029] Haydon PG (2001). Glia: Listening and talking to the synapse. Nat Rev Neurosci.

[pbio-0050139-b030] Kang J, Jiang L, Goldman SA, Nedergaard M (1998). Astrocyte-mediated potentiation of inhibitory synaptic transmission. Nat Neurosci.

[pbio-0050139-b031] Ullian EM, Sapperstein SK, Christopherson KS, Barres BA (2001). Control of synapse number by glia. Science.

[pbio-0050139-b032] Herman LH, Hurley S, Nudds M (2006). Intelligence and rational behaviour in the bottlenosed dolphin. Rational animals?.

[pbio-0050139-b033] Herman LM, Gordon JA (1974). Auditory delayed matching in the bottlenosed dolphin. J Exp Anal Behav.

[pbio-0050139-b034] Herman LM, Hovancik JR, Gary JD, Bradshaw GL (1989). Generalization of visual matching by a bottlenosed dolphin (Tursiops truncatus): Evidence for invariance of cognitive performance with visual or auditory materials. J Exp Psych: Anim Behav Proc.

[pbio-0050139-b035] Thompson RKR, Herman LM (1977). Memory for lists of sounds by the bottlenosed dolphin: Convergence of memory processes with humans?. Science.

[pbio-0050139-b036] Thompson RKR, Herman LM (1981). Auditory delayed discriminations by the dolphin: Nonequivalence with delayed matching performance. Animal Learn Behav.

[pbio-0050139-b037] Herman LM, Pack AA, Wood AM (1994). Bottlenosed dolphins can generalize rules and develop abstract concepts. Mar Mamm Sci.

[pbio-0050139-b038] Mercado EM, Killebrew DA, Pack AA, Macha IVB, Herman LM (2000). Generalization of same-different classification abilities in bottlenosed dolphins. Behav Proc.

[pbio-0050139-b039] Herman LM, Morrel-Samuels P, Pack AA (1990). Bottlenosed dolphin and human recognition of veridical and degraded video displays of an artificial gestural language. J Exp Psych: Gen.

[pbio-0050139-b040] Herman LM, Richards DG, Wolz JP (1984). Comprehension of sentences by bottlenosed dolphins. Cognition.

[pbio-0050139-b041] Herman LM, Kuczaj SA, Holder MD (1993). Responses to anomalous gestural sequences by a language-trained dolphin: Evidence for processing of semantic relations and syntactic information. J Exp Psychol: Gen.

[pbio-0050139-b042] Reiss D, McCowan B (1993). Spontaneous vocal mimicry and production by bottlenosed dolphins (Tursiops truncatus): Evidence for vocal learning. J Comp Psychol.

[pbio-0050139-b043] Richards D, Wolz J, Herman LM (1984). Vocal mimicry of computer-generated sounds and vocal labeling of objects by a bottlenose dolphin, Tursiops truncatus. J Comp Psychol.

[pbio-0050139-b044] Herman LH, Dautenhahn K, Nehaniv CL (2002). Vocal, social, and self-imitation by bottlenosed dolphins. Imitation in animals and artifacts.

[pbio-0050139-b045] Hooper S, Reiss D, Carter M, McCowan B (2006). Importance of contextual saliency on vocal imitation by bottlenose dolphins. Int J Comp Psychol.

[pbio-0050139-b046] Whiten A (2001). Imitation and cultural transmission in apes and cetaceans. Behav Brain Sci.

[pbio-0050139-b047] Herman LM, Abichandani SL, Elhajj AN, Herman EYK, Sanchez JL (1999). Dolphins (Tursiops truncatus) comprehend the referential character of the human pointing gesture. J Comp Psychol.

[pbio-0050139-b048] Pack AA, Herman LM (2004). Dolphins (Tursiops truncatus) comprehend the referent of both static and dynamic human gazing and pointing in an object choice task. J Comp Psychol.

[pbio-0050139-b049] Tschudin A, Call J, Dunbar RIM, Harris G, van der Elst C (2001). Comprehension of signs by dolphins (Tursiops truncatus). J Comp Psychol.

[pbio-0050139-b050] Pack AA, Herman LM (2007). The dolphin's (Tursiops truncatus) understanding of human gaze and pointing: Knowing what and where. J Comp Psychol.

[pbio-0050139-b051] Xitco MJ, Roitblat HL (1996). Object recognition through eavesdropping: Passive echolocation in bottlenose dolphins. Anim Learn Behav.

[pbio-0050139-b052] Xitco MJ, Gory JD, Kuczaj SA (2001). Spontaneous pointing by bottlenose dolphins (Tursiops truncatus). Anim Cogn.

[pbio-0050139-b053] Xitco JJ, Gory JD, Kuczaj SA (2004). Dolphin pointing is linked to the attentional behavior of a receiver. Anim Cogn.

[pbio-0050139-b054] Reiss D, Marino L (2001). Self-recognition in the bottlenose dolphin: A case of cognitive convergence. Proc Natl Acad Sci U S A.

[pbio-0050139-b055] Plotnik JM, de Waal FBM, Reiss D (2006). Self-recognition in an Asian elephant. Proc Natl Acad Sci U S A.

[pbio-0050139-b056] McCowan B, Marino L, Vance E, Walke L, Reiss D (2000). Bubble ring play of bottlenose dolphins: Implications for cognition. J Comp Psychol.

[pbio-0050139-b057] Mercado E, Murray SO, Uyeyama RK, Pack AA, Herman LM (1998). Memory for recent actions in the bottlenosed dolphin (Tursiops truncatus): Repetition of arbitrary behaviors using an abstract rule. Anim Learn Behav.

[pbio-0050139-b058] Mercado E, Uyeyama RK, Pack AA, Herman LM (1999). Memory for action events in the bottlenosed dolphin. Anim Cogn.

[pbio-0050139-b059] Herman LM, Matus DS, Herman EY, Ivancic M, Pack AA (2001). The bottlenosed dolphin's (Tursiops truncatus) understanding of gestures as symbolic representations of its body parts. Anim Learn Behav.

[pbio-0050139-b060] Smith JD, Schull J, Strote J, McGee K, Egnor R (1995). The uncertain response in the bottlenose dolphin (Tursiops truncatus). J Exp Psychol: Gen.

[pbio-0050139-b061] Connor RC, Wells R, Mann J, Read A, Mann J, Connor RC, Tyack P, Whitehead H (2000). The bottlenose dolphin: social relationships in a fission-fusion society. Cetacean societies: Field studies of whales and dolphins.

[pbio-0050139-b062] Baird R, Mann J, Connor RC, Tyack P, Whitehead H (2000). The killer whale: Foraging specializations and group hunting. Cetacean societies: Field studies of whales and dolphins.

[pbio-0050139-b063] Gazda SK, Connor RC, Edgar RK, Cox F (2005). A division of labour with role specialization in group-hunting bottlenose dolphins (Tursiops truncatus) off Cedar Key, Florida. Proc R Soc Lond Ser B.

[pbio-0050139-b064] Lusseau D (2006). Why do dolphins jump? Interpreting the behavioural repertoire of bottlenose dolphins (Tursiops sp.) in Doubtful Sound, New Zealand. Behav Proc.

[pbio-0050139-b065] Lusseau D (2007). Evidence for social role in a dolphin social network. Evol Ecol.

[pbio-0050139-b066] Rendell LE, Whitehead H (2001). Culture in whales and dolphins. Behav Brain Sci.

[pbio-0050139-b067] Ford JKB, Ellis GM, Balcomb KC (2000). Killer whales.

[pbio-0050139-b068] Yurk H, de Waal FBM, Tyack PL (2003). Do killer whales have culture. Animal social complexity: Intelligence, culture, and individualized societies.

[pbio-0050139-b069] Herman LM, Tavolga WN, Herman LM (1980). The communication systems of cetaceans. Cetacean behavior: Mechanisms and functions.

[pbio-0050139-b070] Reidenberg JS, Laitman JT (1987). Position of the larynx in Odontoceti (toothed whales). Anat Rec.

[pbio-0050139-b071] Reidenberg JS, Laitman JT (1988). Existence of vocal folds in the larynx of Odontoceti (toothed whales). Anat Rec.

[pbio-0050139-b072] Reidenberg JS, Laitman JT (1994). Anatomy of the hyoid apparatus in Odontoceti (toothed whales): Specializations of their skeleton and musculature compared with those of terrestrial mammals. Anat Rec.

[pbio-0050139-b073] Reidenberg JS, Laitman JT (1999). Identifying the sound source in mysticetes. Eur Res Cetaceans.

[pbio-0050139-b074] Reidenberg JS, Laitman JT (2004). Anatomy of infrasonic communication in baleen whales: Divergent mechanisms of sound generation in mysticetes and odontocetes.

[pbio-0050139-b075] Ferrer I, Cancho R, Lusseau D (2006). Long-term correlations in the surface behavior of dolphins. Europhys Lett.

[pbio-0050139-b076] McCowan B, Hanser SF, Doyle LR (1999). Quantitative tools for comparing animal communication systems: information theory applied to bottlenose dolphin whistle repertoires. Anim Behav.

[pbio-0050139-b077] McCowan B, Doyle LR, Hanser SF (2002). Using information theory to assess the diversity, complexity and development of communicative repertoires. J Comp Psychol.

[pbio-0050139-b078] Ford JKB (1991). Vocal traditions among resident killer whales (Orcinus orca) in coastal waters of British Columbia. Can J Zool.

[pbio-0050139-b079] Yurk H, Barrett-Lennard L, Ford JKB, Matkin CO (2002). Cultural transmission within maternal lineages: Vocal clans in resident killer whales in southern Alaska. Anim Behav.

[pbio-0050139-b080] Deecke VB, Ford JKB, Spong P (2000). Dialect change in resident killer whales: implications for vocal learning and cultural transmission. Anim Behav.

[pbio-0050139-b081] Rendell LE, Whitehead H (2003). Vocal clans in sperm whales (Physeter macrocephalus). Proc Biol Sci.

[pbio-0050139-b082] Deecke VB, Slater PJB, Ford JKB (2002). Selective habituation shapes acoustic predator recognition in harbour seals. Nature.

[pbio-0050139-b083] Janik VM, Sayigh LS, Well RS (2006). Signature whistle shape conveys identity information to bottlenose dolphins. Proc Natl Acad Sci U S A.

[pbio-0050139-b084] Guinet C, Bouvier J (1995). Development of intentional stranding hunting techniques in killer whale (Orcinus orca) calves at Crozet archipelago. Can J Zool.

[pbio-0050139-b085] Foote AD, Griffin RM, Howitt D, Larsson L, Miller PJO (2007). Killer whales are capable of vocal learning. Biol Lett.

[pbio-0050139-b086] Connor RC, Smolker R, Bejder L (2006). Synchrony, social behavior and alliance affiliation in Indian Ocean bottlenose dolphins, Tursiops aduncus. Anim Behav.

[pbio-0050139-b087] Reader SM, Laland KN (2002). Social intelligence, innovation, and enhanced brain size in primates. Proc Natl Acad Sci U S A.

[pbio-0050139-b088] Lefebvre L, Nicolakakis N, Boire D (2002). Tools and brains in birds. Behav.

[pbio-0050139-b089] Krûtzen M, Mann J, Heithaus MR, Connor RC, Bejder L, Sherwin WB (2005). Cultural transmission of tool use in bottlenose dolphins. Proc Natl Acad Sci U S A.

[pbio-0050139-b090] Magnan A (1934). Le Vol des Insects.

